# Preserved *SCN4B* expression is an independent indicator of favorable recurrence-free survival in classical papillary thyroid cancer

**DOI:** 10.1371/journal.pone.0197007

**Published:** 2018-05-03

**Authors:** Yanping Gong, Jing Yang, Wenshuang Wu, Feng Liu, Anping Su, Zhihui Li, Jingqiang Zhu, Tao Wei

**Affiliations:** Thyroid and Parathyroid Surgery Center, West China Hospital, Sichuan University, Chengdu, Sichuan, China; University of South Alabama Mitchell Cancer Institute, UNITED STATES

## Abstract

Voltage-gated sodium channel β subunits (encoded by *SCN1B* to *SCN4B* genes) have been demonstrated as important multifunctional signaling molecules modulating cellular processes such as cell adhesion and cell migration. In this study, we aimed to explore the expression profiles of *SCN4B* in papillary thyroid cancer (PTC) and its prognostic value in terms of recurrence-free survival (RFS) in classical PTC. In addition, we also examined the potential effect of DNA methylation on its expression. A retrospective study was performed by using data from available large databases, including the Gene Expression Omnibus (GEO) datasets and the Cancer Genome Atlas (TCGA)-Thyroid Cancer (THCA). Results showed that *SCN4B* is downregulated at both RNA and protein level in PTC compared with normal thyroid tissues. Preserved *SCN4B* expression was an independent indicator of favorable RFS in patients with classical PTC, no matter as categorical variables (HR: 0.243, 95%CI: 0.107–0.551, *p* = 0.001) or as a continuous variable (HR: 0.684, 95%CI: 0.520–0.899, *p* = 0.007). The methylation status of one CpG site (Chr11: 118,022,316–318) in *SCN4B* DNA had a moderately negative correlation with *SCN4B* expression in all PTC cases (Pearson’s r = -0.48) and in classical PTC cases (Pearson’s r = -0.41). In comparison, *SCN4B* DNA copy number alterations (CNAs) were not frequent and might not influence its mRNA expression. In addition, no somatic mutation was found in *SCN4B* DNA. Based on these findings, we infer that preserved *SCN4B* expression might independently predict favorable RFS in classical PTC. Its expression might be suppressed by DNA hypermethylation, but is less likely to be influenced by DNA CNAs/mutations.

## Introduction

Voltage-gated sodium channels are integral membrane proteins that constitute one large pore-forming principal α subunit and one or two smaller transmembrane β subunits as auxiliary (encoded by *SCN1B* to *SCN4B* genes) [1, 2]. Although the β subunits were firstly identified as auxiliary subunits modulating the gating, kinetics, and localization of the ion channel pore, there are emerging studies showed that they are also important multifunctional signaling molecules regulating cell adhesion, cell migration, differentiation, endosome acidification, phagocytosis and podosome formation, with or without the presence of pore-forming α subunit [2, 3]. Some recent studies found that the sodium channel β subunits are dysregulated in oncogenic processes. *SCN1B* expression is decreased in highly metastatic breast cancer cells lines [4], but is increased in highly metastatic prostate cancer cell lines [5]. In breast cancer cells, decreased SCN4B protein expression correlates with high-grade primary and metastatic breast tumors and is also associated with enhanced breast cancer cell migration, invasiveness and metastatic spreading [6].

Papillary Thyroid Cancer (PTC) is the dominant form of thyroid cancer, which is usually indolent in progression [7, 8]. The standard treatment of PTC is total thyroidectomy or hemithyroidectomy with following radioiodine ablation and thyrotropin suppression in appropriately selected cases. Patients after these treatments generally have a favorable prognosis, with a 10-year survival rate over 95% [8]. However, disease recurrence and/or distant metastasis were observed in about 5–20% of the patients, which lead to aggressive and lethal outcomes [7, 9]. PTC consists of several histological subtypes, such as classical/usual PTC, follicular PTC, and tall-cell PTC, among which the classical PTC is the most prevalent subtype. These subtypes have different biological behaviors and may have different clinical implications [7, 9]. Therefore, it is meaningful to explore the biomarker of recurrence in different histological subtypes.

In this study, by using data from the Cancer Genome Atlas (TCGA)-Thyroid Cancer (THCA), we performed a retrospective study to explore the expression profiles of *SCN4B* in PTC and its prognostic value in terms of recurrence-free survival (RFS) in classical PTC. In addition, we also examined the potential effect of DNA methylation on its expression.

## Materials and methods

### Secondary analysis of microarray data in GEO datasets

In the Gene Expression Omnibus (GEO) datasets, one previous Affymetrix Human Genome U133 Plus 2.0 Array (GSE3678) analyzed the gene expression profiles of 7 PTC samples compared to 7 paired normal samples. The raw SOFT data file of this array was downloaded and reanalyzed to identify the expression profile of sodium channel subunits.

### SCN4B Immunohistochemistry (IHC) staining

SCN4B IHC staining in normal thyroid and PTC tissues was examined in the Human Protein Atlas (http://www.proteinatlas.org/) [10, 11], which is an online tool for genome-wide analysis of the human proteins.

### Retrospective analysis using data from TCGA-THCA

The association between *SCN4B* expression and the clinicopathological parameters and RFS in PTC patients was studied by performing a retrospective analysis using the level 3 data of TCGA-THCA. Data mining was performed as introduced by one previous study [12]. According to the description by TCGA, the pathological assessment of the biospecimens was performed a board-certified pathologist to ensure the accuracy [13]. Tumors were classified as the follicular variant if it were 99% follicular patterned, and as the tall cell variant if it had 50% or greater tall cell features [13]. In brief, the original data, including sample type, age at initial pathologic diagnosis, histological types, gender, pathological stage, lymph nodal invasion, residual tumors, the history of radiation therapy, recurrence status and RFS in days were obtained by using the UCSC Xena Browser (https://xenabrowser.net/). In this patient cohort, the tumor tissue from 505 PTC cases (358 classical/usual cases, 102 follicular cases, 36 tall cell cases and 4 cases not specified) and 59 normal thyroid tissues were subjected to RNA-seq (by IlluminaHiSeq). 358 out the 505 PTC cases belong to classical/usual histological subtype. 348 out of the 358 classical PTC cases had RFS data recorded and were subjected to survival analysis. The flowchart showing the inclusion of patients was given in S1 Fig. Kaplan-Meier curves of RFS were generated by using GraphPad Prism 6.0 (GraphPad Inc.).

The *SCN4B* DNA methylation data (Illumina 450k infinium methylation beadchip), Gene-level thresholded GISTIC2-processed DNA copy number alterations (CNAs) data, as well as DNA mutation data (SNPs and small insertions and deletions (INDELs)) were downloaded to investigate the potential mechanisms of *SCN4B* dysregulation in PTC.

### Statistical analysis

Data were reported as means ± standard deviations (SDs). Statistical analysis was performed by using Prism 6.0 or SPSS 19.0 software package (SPSS Inc.). Welch's unequal variances t-test was applied to compare the difference in *SCN4B* expression between groups with different clinicopathological parameters. Receiver operating characteristic (ROC) analysis for recurrence detection was applied to identify the best cut-off (Youden index) for *SCN4B* expression in survival analysis.

Chi-square tests were performed to compare the association between *SCN4B* expression and the clinicopathological parameters. Kaplan-Meier curves of RFS was generated using GraphPad Prism 6.0. Patients were grouped by setting the Youden index as the cut-off. Log-rank test was performed to assess the significance of the difference between the survival curves. Univariate and multivariate Cox regression models were used to evaluate the independent prognostic value of *SCN4B* expression (as either categorical variables or a continuous variable) in terms of RFS. *p*<0.05 was considered statistically significant.

## Results

### *SCN4B* was downregulated in PTC compared with normal thyroid tissues

**Fig 1:**
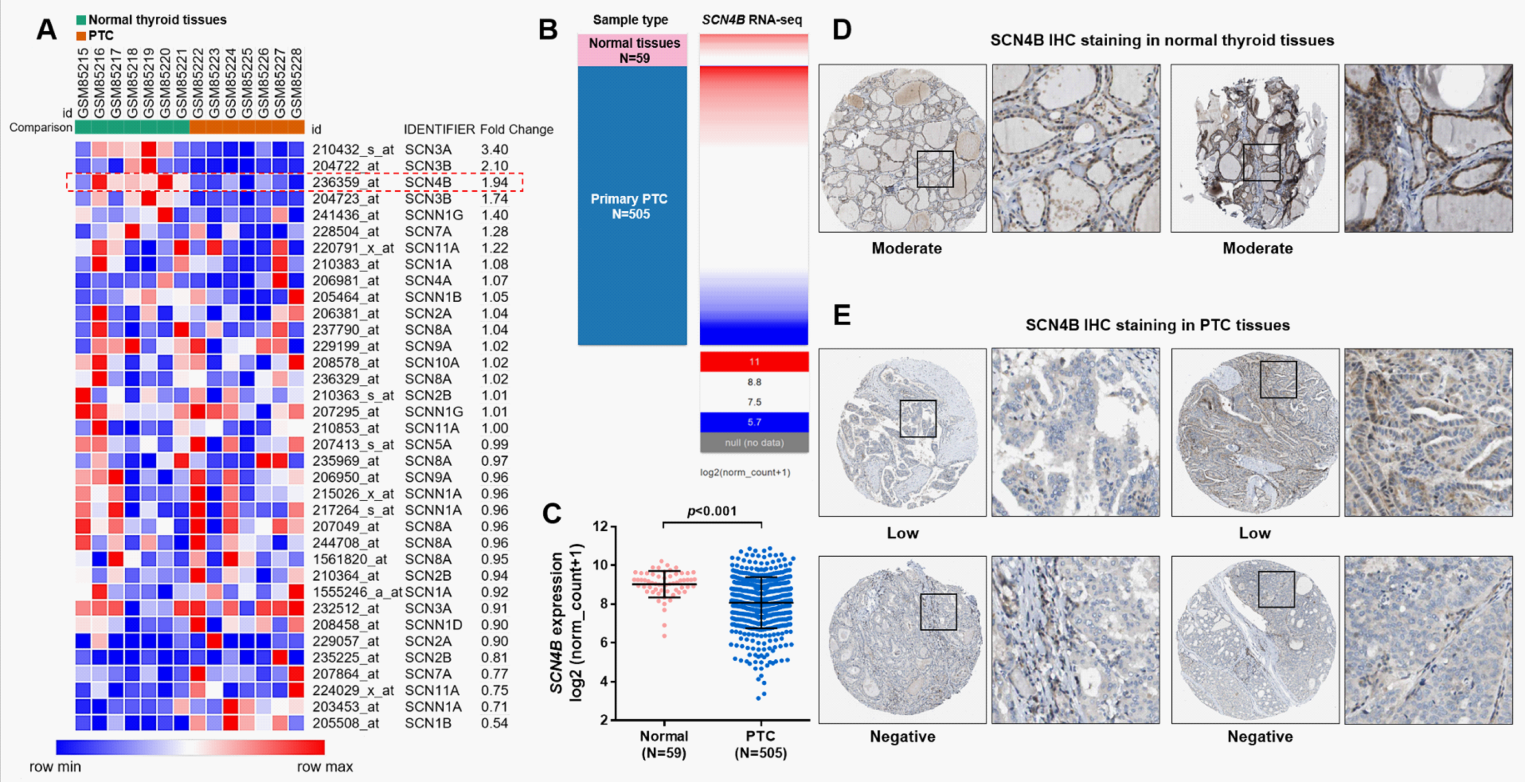
*SCN4B* is downregulated in PTC tissues compared with normal thyroid tissues. **A.** Heatmap of the expression of sodium channel subunits between PTC and 7 paired normal samples. Results were generated by reanalysis of the raw data of GSE3678. **B-C.** Heatmap (B) and plots chart (C) of *SCN4B* expression in PTC (N = 505) and normal thyroid tissues (N = 59). Data was obtained from TCGA-THCA. **D-E.** Representative SCN4B IHC staining (brown) in normal thyroid tissues (D) and in PTC tissues (E). Image credit: Human Protein Atlas. SCN4B images were obtained from: v18.proteinatlas.org, http://www.proteinatlas.org/ENSG00000177098-SCN4B/tissue/thyroid+gland#img and http://www.proteinatlas.org/ENSG00000177098-SCN4B/pathology/tissue/thyroid+cancer#ihc.

Using the raw data of one previous array (GSE3678), we examined the expression profiles of sodium channel subunits between normal thyroid tissues and PTC tissues (Fig 1A). The heatmap of the array results showed that *SCN4B* was one of the most downregulated sodium channel subunits in PTC compared with normal thyroid tissues (Fig 1A, red dotted frame). To verify this dysregulation, we also examined its expression by using RNA-seq data in TCGA-THCA. In this cohort, 505 primary PTC tissues and 59 normal thyroid tissues were subjected to RNA-seq (Fig 1B). Heatmap and the following comparison showed that *SCN4B* was significantly reduced in PTC tissues (*p*<0.001, Fig 1B and 1C). Then, using human tissue IHC staining results in the HPA, we also examined SCN4B protein expression in normal thyroid and PTC tissues. The staining results indicated that normal thyroid usually had moderate SCN4B expression (Fig 1D). In comparison, among 4 cases of PTC tissues examined, only two cases had low SCN4B staining, while the rest two cases had negative SCN4B expression (Fig 1E). These findings suggest *SCN4B* is downregulated at both RNA and protein level in PTC compared with normal thyroid tissues.

### Decreased *SCN4B* expression was associated with recurrence in classical PTC

**Fig 2:**
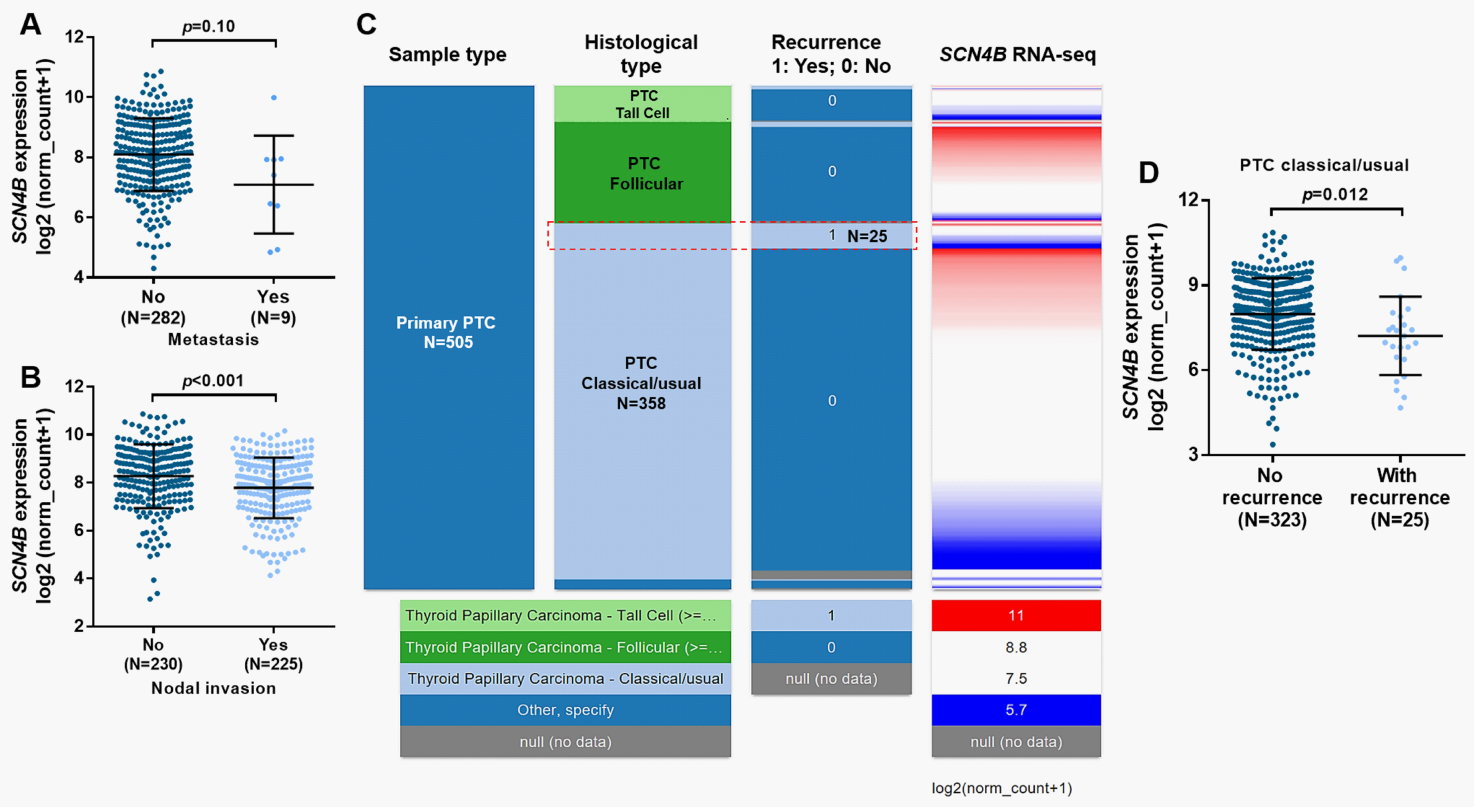
Decreased *SCN4B* expression was associated with recurrence in classical PTC. **A-B.** Plots chart of *SCN4B* expression between the PTC cases with or without metastasis (A) and between the cases with or without lymph nodal invasion (B). **C.** Heatmap showing the correlation between tumor recurrence and *SCN4B* expression in different PTC histological types. **D.** Plots chart of *SCN4B* expression between the classical/usual PTC cases with or without recurrence. Reanalysis was performed by using data from TCGA-THCA.

One previous study reported that *SCN4B* is a metastasis-suppressor gene in breast cancer [6]. In this study, we also examined the association between SCN4B expression and metastasis and lymph nodal invasion in PTC. Results showed that there was no significant difference in *SCN4B* expression between the cases with or without metastasis (Fig 2A). However, the lymph nodal positive cases had substantially decreased *SCN4B* expression compared their counterparts (Fig 2B). Using the survival data in TCGA-THCA, we also examined the recurrence status of different histological PTC cases. Heatmap showed that the classical/usual PTC subtype had the largest proportion of recurrence (Fig 2C, red dotted frame). The cases with recurrence also had significantly decreased *SCN4B* expression (*p* = 0.012, Fig 2D).

### Preserved *SCN4B* expression is an independent indicator of favorable RFS in classical PTC

**Fig 3:**
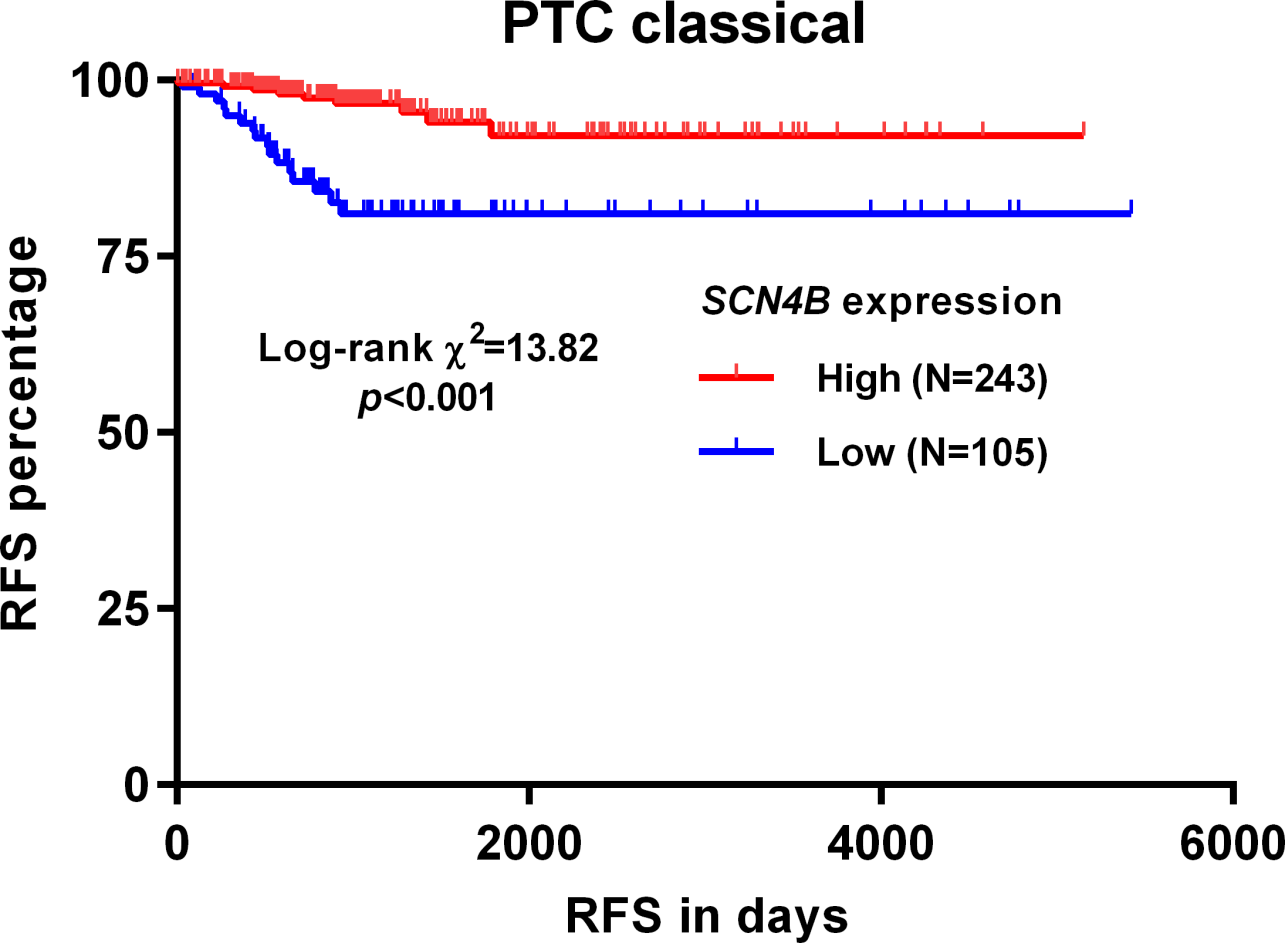
Kaplan-Meier curves of RFS in patients with classical PTC.

By generating Kaplan-Meier curves of RFS in classical PTC patients, we found that high *SCN4B* expression was associated with significantly better RFS (*p*<0.001, Fig 3). The clinicopathological parameters in the high and low *SCN4B* expression groups were summarized in Table 1. Chi-square analysis showed that the high *SCN4B* expression was associated with a significantly lower ratio of nodal invasion (115/227, 50.7% vs. 61/96, 63.5%, *p* = 0.034) and recurrence (9/243, 3.7% vs. 16/105, 15.2%, *p*<0.001) compared with the low *SCN4B* expression group (Table 1). In univariate COX regression analysis, low pathological stage, no residual tumors and high *SCN4B* expression (as categorical variables)/increased *SCN4B* expression (as a continuous variable) were associated with favorable RFS (Tables 2 and 3). In the following multivariate analysis, preserved *SCN4B* expression was an independent indicator of favorable RFS, no matter as categorical variables (HR: 0.243, 95%CI: 0.107–0.551, *p* = 0.001) (Table 2) or as a continuous variable (HR: 0.684, 95%CI: 0.520–0.899, *p* = 0.007) (Table 3).

**Table 1:** The association between *SCN4B* expression and the clinicopathological parameters in patients with classical PTC.

Parameters		*SCN4B* expression		χ^2^	*p*
		High (N = 243)	Low (N = 105)		
**Age (Mean ± SD)**		46.61±15.84	45.71±16.95	N.A.	0.64
**Gender**	Female	175	77	0.06	0.80
	Male	68	28		
**Pathological Stage**	III/IV	75	36	0.40	0.53
	I/II	168	69		
**Nodal invasion**	No	112	35	4.51	0.034
	Yes	115	61		
	NX/No data	16	9		
**Residual tumors**	R0	183	81	0.004	0.95
	R1/R2	30	13		
	RX/no data	30	11		
**Radiation therapy**	No	90	40	0.025	0.87
	Yes	145	62		
	No data	8	3		
**Recurrence status**	No	234	89	14.63	<0.001
	Yes	9	16		

NX: Regional lymph nodes cannot be assessed; R0: No residual tumor; R1: Microscopic residual tumor; R2: Macroscopic residual tumor; RX: The presence of residual tumor cannot be assessed.

**Table 2:** Univariate and multivariate analysis of RFS in patients with classical PTC (*SCN4B* expression as categorical variables).

Parameters	Univariate analysis				Multivariate analysis			
	*p*	HR	95%CI (lower/upper)		*p*	HR	95%CI (lower/upper)	
**Age** (Continuous)	0.276	1.013	0.990	1.037				
**Gender** Female *vs*. Male	0.501	0.749	0.323	1.737				
**Clinical stage** III/IV *vs*. I/II	0.035	2.329	1.061	5.113	0.139	1.872	0.816	4.296
**Nodal invasion** No *vs*. Yes	0.843	0.920	0.403	2.099				
**Residual tumors** No *vs*. Yes	0.014	0.332	0.137	0.800	0.052	0.398	0.157	1.009
***SCN4B* expression** (High *vs*. Low)	0.001	0.240	0.106	0.544	0.001	0.243	0.107	0.551

**Table 3:** Univariate and multivariate analysis of RFS in patients with classical PTC (*SCN4B* expression as a continuous variable).

Parameters	Univariate analysis				Multivariate analysis			
	*p*	HR	95%CI (lower/upper)		*p*	HR	95%CI (lower/upper)	
**Clinical stage** III/IV *vs*. I/II	0.035	2.329	1.061	5.113	0.115	1.943	0.850	4.444
**Residual tumors** No vs. Yes	0.014	0.332	0.137	0.800	0.064	0.417	0.165	1.051
***SCN4B* expression** (Continuous)	0.005	0.681	0.522	0.889	0.007	0.684	0.520	0.899

### DNA hypomethylation might be a mechanism of decreased *SCN4B* expression in PTC

**Fig 4:**
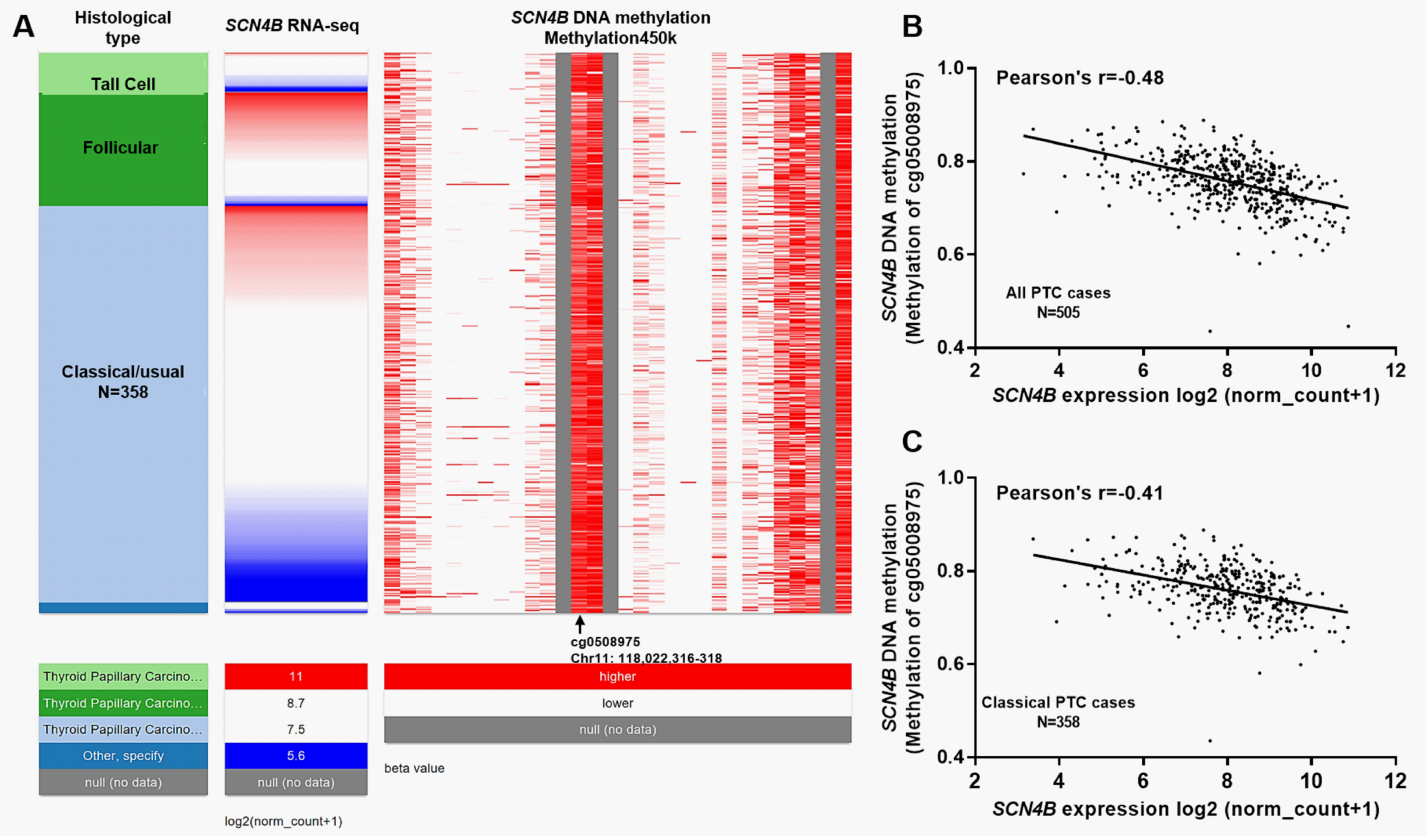
DNA hypomethylation might be a mechanism of decreased *SCN4B* expression in PTC. **A.** Heatmap showing the correlation between *SCN4B* expression and its DNA methylation (Methylation 450k) in different subtypes of PTC. **B-C.** Regression analysis of the correlation between *SCN4B* expression and its DNA methylation in all PTCs (B) and classical subtypes (C).

In Illumina 450k infinium methylation beadchip, the methylation status of 27 CpG sites in *SCN4B* DNA was measured. By comparing SCN4B expression and its DNA methylation, we found that the methylation status of one CpG site (Chr11: 118,022,316–318) was negatively correlated with *SCN4B* expression (Fig 4A). Regression analysis confirmed a moderately negative correlation in all PTC cases (Pearson’s r = -0.48) (Fig 4B). In classical PTC cases, this negative correlation was also confirmed (Pearson’s r = -0.41) (Fig 4C). Then, we also examined the association between *SCN4B* RNA expression and its DNA CNAs/mutations. Results showed that among 505 primary PTC cases, 497 cases had CNAs measured, while only 14 cases had CNAs (9 heterozygous loss (-1) and 5 low-level copy gain (+1)) (S2A and S2B Fig). These alterations did not influence *SCN4B* expression (S2A and S2B Fig). Besides, no somatic mutation was found in *SCN4B* DNA (S2A Fig).

## Discussion

There are emerging studies showed that dysregulated sodium channel β subunits are implicated in multiple types of cancer. In mouse model bearing implanted prostate tumor, *SCN1B* expression is associated with enhanced growth rate and size, as well as decreases in survival rates [5]. In breast cancer, enforced *SCN1B* expression could promote pathological growth and cellular dissemination, including metastasis to both lung and liver [14]. In cervical cancer tissues, *SCN3B* mRNA level is increased, whereas *SCN1B*, *SCN2B* and *SCN4B* mRNA levels are decreased [15]. SCN3B is induced in mouse embryonic fibroblasts by DNA damage in a p53-dependent manner and mediates a p53-dependent apoptotic pathway [16]. In breast cancer cells, reduced SCN4B expression is associated with increased RhoA activity, enhanced cell migration and invasiveness, primary tumor growth and metastatic spreading, via promoting the acquisition of an amoeboid-mesenchymal hybrid phenotype [6]. These results suggest that the expression and functional role of sodium channel β subunits might be tissue specific.

In this study, by using available large databases, we examined the expression profiles of sodium channel subunits in PTC and found that *SCN4B* was significantly downregulated at both mRNA and protein levels in PTC compared with normal thyroid tissues. In addition, we also observed that the PTC cases with lymph nodal invasion had significantly lower *SCN4B* expression. In classical PTC subtype, we confirmed the association between decreased *SCN4B* expression and the risk of recurrence. PTC patients usually have long-term overall survival and the primary goal of surgery is to minimize the risk of local recurrence and distant metastasis [17]. Therefore, we decided to further investigate the potential prognostic value of *SCN4B* expression in terms of RFS in the classical subtype. By generating Kaplan-Meier curves of RFS, we found that the high *SCN4B* expression group had significantly better RFS. The following univariate and multivariate analysis confirmed that preserved *SCN4B* expression was an independent indicator of favorable RFS in patients with classical PTC, no matter as categorical variables (HR: 0.243, 95%CI: 0.107–0.551, *p* = 0.001) or as a continuous variable (HR: 0.684, 95%CI: 0.520–0.899, *p* = 0.007). These findings suggest that *SCN4B* might be a promising prognostic biomarker in classical PTC.

Genetic mutation and epigenetic alteration (such as DNA hypomethylation) are important mechanisms leading to suppressed transcription of some important tumor suppressors in cancers, including PTC [18, 19]. For example, *RASAL1* is a major tumor suppressor gene in thyroid cancer, which is frequently inactivated by hypermethylation and mutations [20]. *ZIC1* is also a tumor suppressor gene in thyroid cancer by blocking the activities of the PI3K/Akt and MAPK signaling pathways and the transcription of transcription factor FOXO3a [21]. However, it is frequently inactivated by promoter hypermethylation [21]. *CDH1* and *SCL5A8* promoter methylation are also associated with the carcinogenesis of thyroid tumor [22]. In this study, by examining the methylation status of 27 CpG sites in *SCN4B* DNA, we found that the methylation status of one CpG site (Chr11: 118,022,316–318) had a moderately negative correlation with *SCN4B* expression in all PTC cases (Pearson’s r = -0.48) and in classical PTC cases (Pearson’s r = -0.41). In comparison, *SCN4B* DNA CNAs were not frequent and might not influence its mRNA expression. In addition, no somatic mutation was found in *SCN4B* DNA. These findings suggest that DNA hypermethylation might be an important mechanism of suppressed *SCN4B* expression in PTC.

## Conclusion

In summary, findings in the study showed that *SCN4B* is downregulated in PTC compared with normal thyroid tissues. Preserved *SCN4B* expression might independently predict favorable RFS in classical PTC. Its expression might be suppressed by DNA hypermethylation, but is less likely to be influenced by DNA CNAs/mutations.

## Supporting information

S1 Fig The flowchart showing the inclusion of patients. (JPG)

S2 Fig *SCN4B* DNA CNAs and mutations. **A.** Heatmap showing the correlation between *SCN4B* expression and its DNA CNAs and mutations in different subtypes of PTC. **B.** Plots chart showing *SCN4B* expression in heterozygous loss (-1), copy-neutral (0) and low-level copy gain (+1) groups.
(JPG)
