# Supplementary figures and images for: Preserved SCN4B expression is an independent indicator of favorable recurrence-free survival in classical papillary thyroid cancer
Source: PLoS One. 2018 May 3;13(5):e0197007. doi: 10.1371/journal.pone.0197007 (PMC5933725; doi:10.1371/journal.pone.0197007)

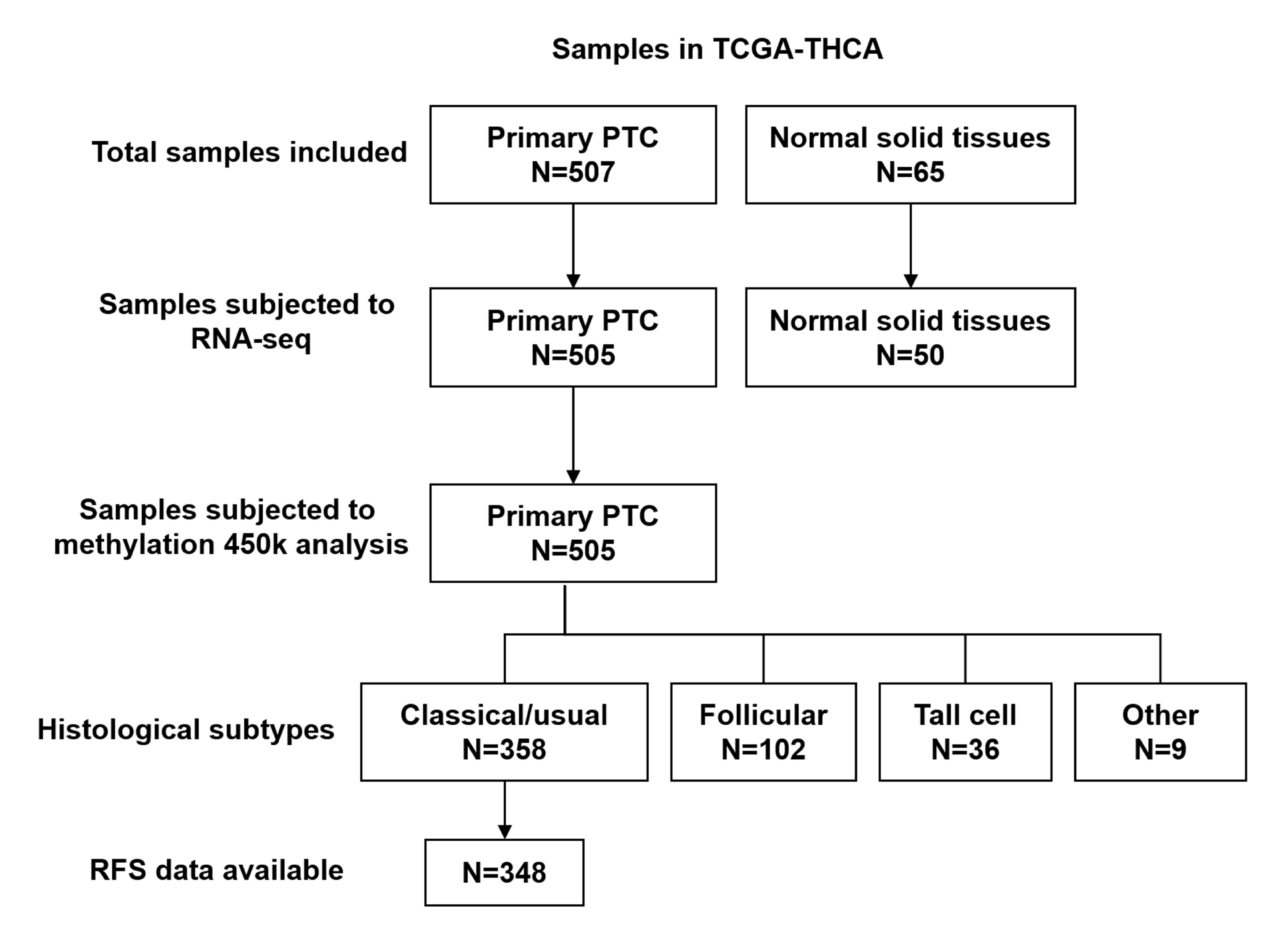

Supplement: S1 Fig — (JPG) [file pone.0197007.s001.jpg]

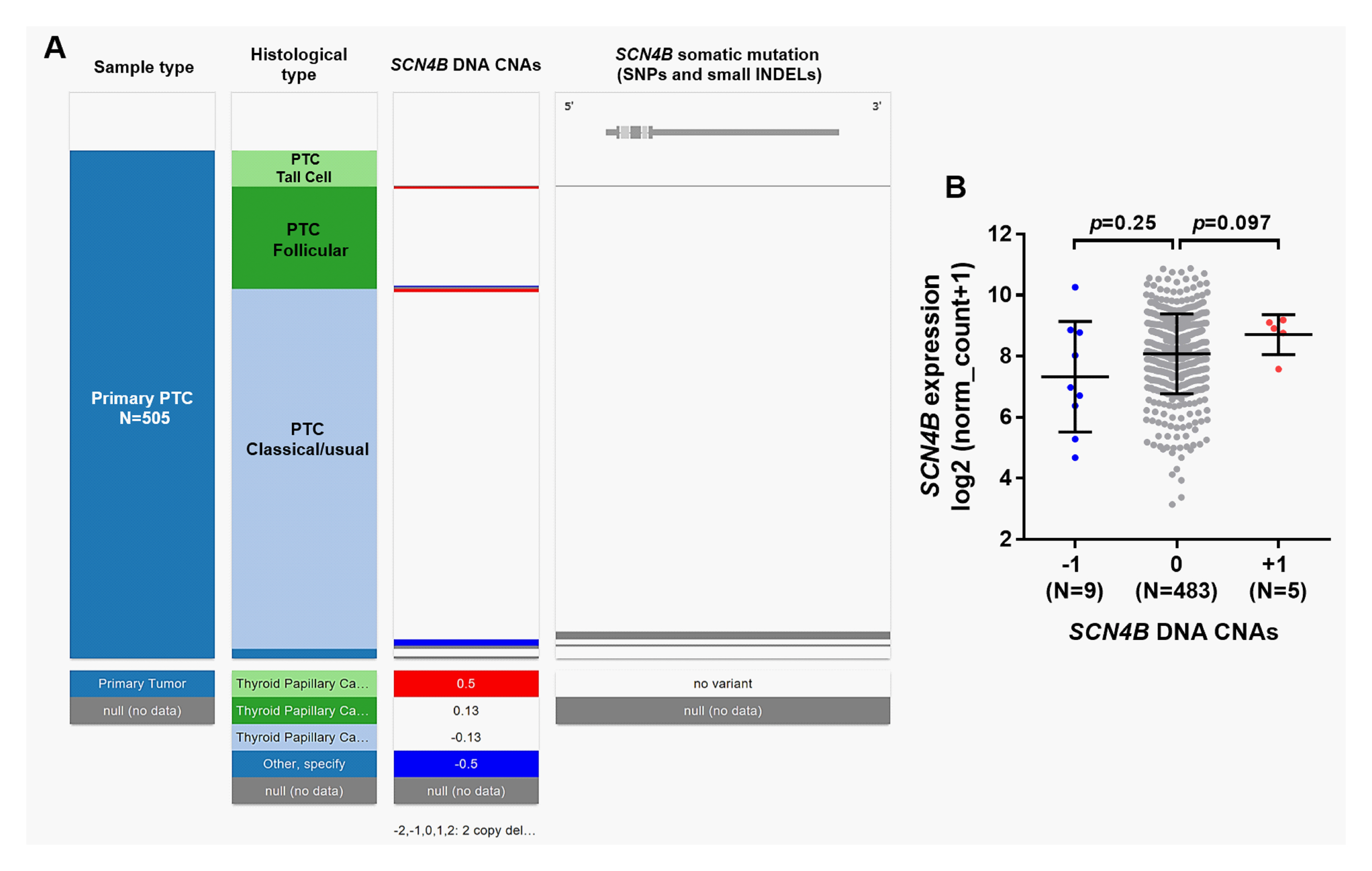

Supplement: S2 Fig — A. Heatmap showing the correlation between SCN4B expression and its DNA CNAs and mutations in different subtypes of PTC. B. Plots chart showing SCN4B expression in heterozygous loss (-1), copy-neutral (0) and low-level copy gain (+1) groups. (JPG) [file pone.0197007.s002.jpg]
